# Bronchoscopy as a supplement to computed tomography in patients with haemoptysis may be unnecessary

**DOI:** 10.3402/ecrj.v3.31802

**Published:** 2016-06-23

**Authors:** Klaus Nielsen, Magnus Gottlieb, Sara Colella, Zaigham Saghir, Klaus R. Larsen, Paul F. Clementsen

**Affiliations:** 1 Department of Respiratory Medicine, Hvidovre University Hospital, Hvidovre, Denmark; 2 Department of Respiratory Medicine, Gentofte University Hospital, Hellerup, Denmark; 3 Department of Pulmonary Rehabilitation, Villa Serena Hospital, Città Sant'Angelo, Italy; 4 Department of Pulmonary Medicine, Bispebjerg University Hospital, Copenhagen, Denmark; 5 Copenhagen Academy for Medical Education and Simulation (CAMES), Rigshospitalet, University of Copenhagen, Copenhagen, Denmark

**Keywords:** haemoptysis, bronchoscopy, computed tomography, sensitivity, specificity

## Abstract

**Background:**

Haemoptysis is a common symptom and can be an early sign of lung cancer. Careful investigation of patients with haemoptysis may lead to early diagnosis. The strategy for investigation of these patients, however, is still being debated.

**Objectives:**

We studied whether the combination of computed tomography (CT) and bronchoscopy had a higher sensitivity for malignant and non-malignant causes of haemoptysis than CT alone.

**Methods:**

The study was a retrospective, non-randomised, two-centre study and included patients who were referred from primary care for the investigation of haemoptysis.

**Results:**

A total of 326 patients were included in the study (mean age 60.5 [SD 15.3] years, 63.3% male). The most common aetiologies of haemoptysis were cryptogenic (52.5%), pneumonia (16.3%), emphysema (8.0%), bronchiectasis (5.8%) and lung cancer (4.0%). In patients diagnosed with lung cancer, bronchoscopy, CT and the combination of bronchoscopy and CT had a sensitivity of 0.61, 0.92 (*p*<0.05) and 0.97 (*p*=0.58), respectively. In patients with non-malignant causes of haemoptysis, most aetiologies were diagnosed by CT and comprised mainly pneumonia, emphysema and bronchiectasis. Bronchoscopy did not reveal these conditions and the sensitivity to these conditions was not increased by combining CT and bronchoscopy.

**Conclusions:**

CT can stand alone as a diagnostic workup for patients with haemoptysis referred to an outpatient clinic. Bronchoscopy does not identify any malignant aetiologies not already diagnosed by CT. Combining the two test modalities does not result in a significant increase in sensitivity for malignant or non-malignant causes of haemoptysis.

On a global scale, lung cancer was the most frequently diagnosed cancer and the cancer causing most deaths among men in 2014 (1). The prognosis is poor and early diagnosis is crucial to allow curative treatment. Haemoptysis may be an early symptom of lung cancer, and careful investigation of patients with this symptom may lead to early diagnosis.

Haemoptysis is a common symptom in clinical practice (2). It is defined as expectoration of blood originating from the tracheobronchial tree or pulmonary parenchyma. Common causes include chronic bronchitis, bronchiectasis, pulmonary embolism, pneumonia, fungal infections, tuberculosis and malignancy (3). In most cases of haemoptysis, the aetiology is benign (4, 5).

The strategy for the investigation of patients with haemoptysis is still under discussion (6). Many studies have analysed the utility of bronchoscopy and computed tomography (CT) in investigating the aetiology and site of bleeding in patients with haemoptysis, but so far, no consensus seems to have been reached. Furthermore, the studies have focused mainly on establishing the best practice for investigating patients with haemoptysis in terms of using either conventional chest X-ray, CT or bronchoscopy.

We decided to investigate the value of the combination of bronchoscopy and CT in unselected consecutive patients presenting with haemoptysis. Our primary end point was the sensitivity of detecting a lung cancer or other thoracic malignancy by the combination of bronchoscopy and CT compared to CT alone. We hypothesized that the combination improves the chance of detecting a lung cancer or other specific diagnoses.

## Materials and methods

### Study population

We included consecutive patients presenting with haemoptysis (ICD-10 diagnosis code R04.2) who were referred to the Department of Respiratory Medicine at either Gentofte Hospital or Bispebjerg Hospital between January 2009 and December 2014. Patients older than 16 years were included if both CT and bronchoscopy were performed, irrespective of smoking history and comorbidities. Exclusion criteria were previously diagnosed lung cancer or thoracic malignancy or incomplete data.

### Study intervention and design

The study was conducted as a retrospective, two-centre, diagnostic study. The data from the two centres were joined as one cross-section and descriptive statistics were performed including age, gender and pack years. Bronchoscopy including biopsy results and cultures and CT findings were recorded as well as the final diagnosis. Bronchoscopy and CT findings were categorised as either *normal* (i.e. not suggestive of any aetiology of the haemoptysis), *suggestive of other lung pathology* or *suggestive of* malignancy (i.e. representing a possible aetiology of the haemoptysis). The final diagnosis was extracted from the patient charts. When no diagnosis was found, two investigators agreed on a consensus diagnosis based on all investigation results. If no reasonable diagnosis could be established based on the findings, the patient case was categorised as having no final diagnosis.

Both centres participating in the study perform CT and bronchoscopy in parallel in patients with haemoptysis irrespective of the result of the individual tests. Only serial testing is done when the clinical suspicion is low or when a patient refuses either CT or bronchoscopy.

### Statistical analysis

Data were presented as frequencies and/or mean±standard deviation (SD). Test sensitivities were compared using the chi-squared test with a significance level of 0.05. Test characteristics for bronchoscopy, CT and the combination of the two were calculated and included sensitivity and specificity. Combined sensitivity for the two parallel tests were calculated using the formula: *Sensitivity of Test A+Sensitivity of Test B – (Sensitivity of Test A x Sensitivity of Test B*), whereas the combined specificity was calculated as *Specificity of A x Specificity of B*.

Data analysis was performed in *Statistical Package for the Social Sciences* (SPSS, IBM Corporation, Version 20.0, Armonk, NY, USA).

The study was conducted in accordance with the Committee on Biomedical Research Ethics for The Capital Region of Denmark (ID 150008368) who required no formal approval process for this kind of study and the Danish Data Protection Agency (ID HGH-2015-001).

## Results

We included 326 patients with haemoptysis who had all undergone both bronchoscopy and CT. The majority were male and current or former smokers (Table 1). In 46 patients (14.1%), pulmonary embolism was suspected and a contrast CT was performed. The remaining patients (85.9%) received a conventional low dose, 5-mm slice CT. Conventional white light bronchoscopy was used in all patients.

**Table 1:** Baseline characteristics of the study population

	*N* (%)
Total number of patients included	326 (100.0)
Male	206 (63.2)
Age, years (mean±SD)	60.5±15.3
Smoking history	
Current smokers	127 (39.0)
Former smokers	135 (41.4)
Pack years (mean±SD)	20.6±20.9
Never smokers	64 (19.6)

In most cases, neither the bronchoscopy nor the CT revealed any cause of the hemoptysis and was thus classified as cryptogenic (52.5%). The most frequent final diagnoses in the remaining patients were pneumonia (16.3%), emphysema (8%), bronchiectasis (5.8%), lung cancer (4%), fibrosis (2.5%) and other infections (1.2%) (Table 2).

**Table 2:** Final diagnosis

	*N*	%
Cryptogenic	171	52.5
Pneumonia	53	16.3
Emphysema	26	8.0
Bronchiectasis	19	5.8
Lung cancer	13	4.0
Fibrosis	8	2.5
Other infections	4	1.2
Pulmonary embolus	3	0.9
Anticoagulant treatment	3	0.9
Heart failure	2	0.6
Pleuritis	2	0.6
Mucosal dysplasia^a^	2	0.6
Allergic alveolitis	1	0.3
Metastatic gastrointestinal cancer	1	0.3
Calcifications	1	0.3
Cicatricial infiltration	1	0.3
Desquamative interstitial pneumonia	1	0.3
Oesophageal infection	1	0.3
Hamartoma	1	0.3
Atelectasis	1	0.3
Idiopathic pulmonary fibrosis	1	0.3
Lymphoid interstitial pneumonia	1	0.3
Osler syndrome	1	0.3
Metastatic breast cancer	1	0.3
Tuberculosis	1	0.3
Metastatic hepatic cancer	1	0.3
Rib fracture	1	0.3
Sarcoidosis	1	0.3
Bronchitis	1	0.3
Pneumothorax	1	0.3
Vascular malformation	1	0.3
Tuberculosis sequelae	1	0.3
Total	326	100.0

a Patients with ongoing clinical follow-ups including bronchoscopy.

### CT findings

The CT scan showed no aetiology of haemoptysis in 152 patients (46.6%) (Table 3). In the other 152 patients (46.6%), the scan was suggestive of a non-malignant condition that could explain the haemoptysis, of which the most common conditions were pneumonia, emphysema and bronchiectasis. In 36 cases where the CT scan was suspicious of other lung pathology, no final diagnosis could be established based on the findings. The remaining 22 patients had a scan that suggested malignancy but lung cancer was only confirmed in 12 of these patients. The sensitivity and specificity of detecting lung cancer on CT were 0.92 (CI: 0.64–1.00) and 0.97 (CI: 0.94–0.98), respectively.

**Table 3:** CT findings and final diagnosis

	*N*		%
No explanation of haemoptysis	152		46.6
Suspicious of malignancy	22		6.7
Lung cancer		*12*	
Other		*4*	
No diagnosis		*3*	
Pneumonia		*2*	
Hamartoma		*1*	
Suspicious of other lung pathology	152		46.6
Pneumonia		*38*	
No diagnosis		*36*	
Emphysema		*23*	
Bronchiectasis		*20*	
Cicatricial infiltrates		*4*	
Fibrosis		*4*	
Other		*4*	
Calcifications		*3*	
Radiation sequelae		*3*	
Pulmonary embolus		*3*	
Pleural effusion		*2*	
Vascular malformation		*1*	
Rib fracture		*1*	
DIP		*1*	
LIP		*1*	
Allergic alveolitis		*1*	
Bronchitis		*1*	
IPF		*1*	
Pneumothorax		*1*	
Lung cancer		*1*	
Tuberculosis		*1*	
Atelectasis		*1*	
Sarcoidosis		*1*	

Values in italic specify the diagnoses established by CT.

### Bronchoscopic findings

The bronchoscopy showed no aetiology of haemoptysis in 272 patients (83.4%). In only 43 patients (13.3%), the bronchoscopy was suspicious of other lung pathology thus having a possible non-malignant cause of the haemoptysis where the most frequent aetiologies were pneumonia and bronchitis. Most cases of lower respiratory infections were confirmed by either subsequent sputum culture or microscopy. In three cases of patients with a bronchoscopy suggestive of other lung pathology, no final diagnosis could be established. In 11 patients, the bronchoscopy suggested malignancy but lung cancer was only confirmed in eight of these patients. The sensitivity and specificity of detecting lung cancer on bronchoscopy were 0.61 (CI: 0.32–0.86) and 0.99 (CI: 0.64–1.00), respectively. In one case, the bronchoscopy revealed an abnormal appearance of the mucosa at the carina of the superior bronchus of the right lower lobe and biopsies showed dysplasia. This patient is subject to regular bronchoscopic follow-ups (Table 4).

**Table 4:** Bronchoscopy findings and final diagnosis

	*N*		%
No explanation of haemoptysis	272		83.4
Suspicious of malignancy	11		3.4
Lung cancer		*8*	
Other		*2*	
Pneumonia		*1*	
Suspicious of other lung pathology	43		13.2
Pneumonia		*29*	
No diagnosis		*3*	
Lung cancer		*2*	
Bronchitis		*2*	
Vascular malformation		*1*	
Bronchiectasis		*1*	
Emphysema		*1*	
Radiation therapy sequelae		*1*	
Oedema of mucosa^a^		*1*	
Fibrosis		*1*	
Tuberculosis sequelae and fungal infection		*1*	

a Patients still in follow-up due to mucosal biopsy showing dysplasia. Values in italic specify the diagnoses established by bronchoscopy.

### Findings by combining CT and bronchoscopy

We did not identify any patient where the CT scan missed a malignant condition that was subsequently identified by bronchoscopy. The sensitivity in detecting lung cancer for bronchoscopy, CT and the combination of bronchoscopy and CT was 0.61, 0.92 and 0.97, respectively, the difference in sensitivity being statistically significant for bronchoscopy versus CT and the combination of CT and bronchoscopy (*p*<0.0001), but insignificant for CT versus the combination of CT and bronchoscopy (*p=*0.58) (Table 5). The most common non-malignant aetiologies of haemoptysis being lower airway tract infections, bronchiectasis and emphysema, bronchoscopy rarely provides any additional information not already found on CT other than providing results of sputum culture and microscopy for patients with airway infections.

**Table 5:** Test characteristics for diagnosing lung cancer

	Bronchoscopy		CT		Bronchoscopy and CT	
			
	95% CI		95% CI		95% CI	
Sensitivity	0.61	0.32–0.86	0.92	0.64–1.00	0.97	0.67–0.99
Specificity	0.99	0.94–0.98	0.97	0.94–0.98	0.96	0.94–0.98

## Discussion

In our cohort, most aetiologies of haemoptysis were cryptogenic. The CT scan and bronchoscopy showed no explanation of the haemoptysis in 46 and 83.4% of our patients. Thirteen patients had lung cancer and CT detected 12 whereas bronchoscopy detected only eight. The sensitivity of the combination of CT and bronchoscopy for the detection of lung cancer was slightly higher than CT alone, but the difference was not statistically significant. We did not calculate sensitivities for the detection of other specific diagnoses because the most common non-malignant causes of haemoptysis were not detectable by bronchoscopy.

Bronchoscopy and CT in combination as a diagnostic workup for patients with hemoptysis is recommended by some authors but there is no agreement concerning to what extent they will provide unique or complementary information (7, 8). CT is superior in diagnosing parenchymal abnormalities, but bronchoscopy is superior in diagnosing mucosal abnormalities in the central airways. The capacity of bronchoscopy to localise the site of bleeding seems equivalent to CT, but it is less useful in detecting an underlying disease (9). Conversely, CT is not suitable for detecting preinvasive lesions and early lung cancer. In this case, bronchoscopy is superior to CT, at least when autofluorescence bronchoscopy is used (10). Other bronchoscopic techniques as narrow-band imaging and confocal fluorescence microscopy have recently been developed but their values have not been proven (11, 12). Normally, conventional white light bronchoscopy is the technique used as a routine. Its place in the diagnostic setup in patients with haemoptysis unexplained by chest X-ray is under discussion but the indication seems to be increased when certain risk factors are present (male, aged 50 years or older, smoking history of more than 40 pack years) and in patients with persistent/recurrent haemoptysis (3, 13, 14).

In a study by Revel et al., CT and bronchoscopic findings were reviewed in 80 patients with haemoptysis. CT was more efficient than bronchoscopy for identifying the cause of bleeding (77% vs 8%, respectively), whereas the methods were comparable for identifying the site of bleeding (70% vs 73%, respectively) (9). In a prospective study, CT and bronchoscopy were compared in 91 patients with haemoptysis, and it was found that CT demonstrated all 27 tumours identified using bronchoscopy as well as seven additional lesions (15). Fourteen cases of bronchiectasis were detected by CT alone (15). It was concluded that bronchoscopy should be used initially when there is a strong suspicion of carcinoma. Thirumaran et al. retrospectively examined 270 patients, 90% smokers or former smokers, presenting with hemoptysis and normal chest radiograph and demonstrated respiratory tract malignancy in 9.6%, 96% of these were detected by CT. These studies suggest that in risk patients with haemoptysis unexplained by chest X-ray, CT should be the first choice followed by bronchoscopy in selected cases, but there is no clear conclusion to be drawn (16).

The recent guidelines from British Thoracic Society recommend to consider bronchoscopy after a normal CT if the patient is at high risk for lung carcinoma or if the haemoptysis continues (17). The Danish Lung Cancer Group (DLCG) recommend in their guidelines that CT and bronchoscopy should be performed in patients who are smokers and 40 years of age or older and who present with haemoptysis for more than 1 week even if the chest radiograph is normal (13, 18).

We found that no additional lung cancer is detected by combining CT and bronchoscopy in patients presenting with haemoptysis compared to CT alone. The sensitivity for detecting a cancer for the combination of CT and bronchoscopy was 0.97 versus 0.92 for CT alone, the difference, however, being insignificant (*p*=0.58). Our findings are supported in a very recent publication by Bønløkke et al. This study included 269 patients with haemoptysis who had all undergone CT and bronchoscopy. Sixteen patients were diagnosed with lung cancer. In all of these patients, a lung tumour was seen on chest CT. No additional cases were found during bronchoscopy (19).

Most non-malignant aetiologies of haemoptysis in our study comprised bronchiectasis, emphysema and lower airway tract infections. The sensitivity of these conditions on CT is 1.00 and combining CT and bronchoscopy in these cases will not provide a better diagnostic yield as the sensitivity will remain 1.00. However, the specificity will be lower for the combination because the probability of having a true negative test result for both tests will be lower.

Many similar studies have reported a higher prevalence of tuberculosis among patients presenting with haemoptysis (16). Most of these patients are identified by the combination of CT or X-ray and bronchoscopy in combination with sputum analysis. We only identified one such patient and this might be explained by a difference in the overall prevalence of tuberculosis in our population compared to that of studies made in other countries. The prevalence of lung cancer in our study and other similar studies is comparable (7); however, the study by Uzun et al. found a prevalence of lung cancer of 29.7% in a Turkish population of 178 individuals (20). We found a prevalence of only 4% which is surprising given the fact that the mean age and smoking history were comparable. The reason for this difference is most likely due to the different ways of referring a patient with haemoptysis for further workup by the primary healthcare system.

The patients in our cohort were referred primarily from primary care for an outpatient workup and presented with mild to moderate hemoptysis. In no cases did we observe ongoing bleeding in the bronchial tree that needed endobronchial hemostasis to be performed. Furthermore, we did not include patients with severe haemoptysis in our study. These latter patient categories are generally admitted through the emergency department and referred directly to a department of thoracic surgery where they may require therapeutic bronchoscopy or invasive radiology with coiling (9).

Our findings may not be applicable to all centres investigating haemoptysis. Countries with a higher incidence of lung cancer or healthcare systems substantially different from ours might have a higher fraction of lung cancer cases that could be identified during bronchoscopy. Furthermore, countries with a higher incidence of tuberculosis might benefit from combining CT and bronchoscopy in patients with haemoptysis as bronchoscopy will increase the chance of detecting the disease due to the finding of acid-fast bacilli in the bronchial lavage fluid.

## Conclusions

Our results suggest that in patients presenting to an outpatient clinic for the investigation of haemoptysis, CT scan can stand alone as a diagnostic workup. Bronchoscopy does not identify any malignant aetiologies not already diagnosed by CT. Combining the two tests does not result in a significant increase in sensitivity for neither lung cancer nor non-malignant causes of haemoptysis. Bronchoscopy only provides additional information in cases where sputum culture or microscopy confirms suspicion of tuberculosis or pneumonia.

## References

[1] Siegel R, Ma J, Zou Z, Jemal A (2014). Cancer statistics, 2014. CA Cancer J Clin.

[2] Johnston RN, Lockhart W, Ritchie MB, Smith DH (1960). Hemoptysis. Br Med J..

[3] Jeudy J, Khan AR, Mohammed T-L, Amorosa JK, Brown K, Dyer DS (2010). ACR appropriateness criteria hemoptysis. J Thorac Imaging..

[4] Hirshberg B, Biran I, Glazer M, Kramer MR (1997). Hemoptysis: etiology, evaluation, and outcome in a tertiary referral hospital. Chest..

[5] Soares Pires F, Teixeira N, Coelho F, Damas C (2011). Hemoptysis–etiology, evaluation and treatment in a university hospital. Rev Port Pneumol..

[6] Larici AR, Franchi P, Occhipinti M, Contegiacomo A, del Ciello A, Calandriello L (2014). Diagnosis and management of hemoptysis. Diagn Interv Radiol..

[7] McGuinness G, Beacher JR, Harkin TJ, Garay SM, Rom WN, Naidich DP (1994). Hemoptysis: prospective high-resolution CT/bronchoscopic correlation. Chest..

[8] Vernhet H, Dogas G, Bousquet C, Durand G, Godard P, Sénac JP (2003). Value of thoracic CT in the management of severe hemoptysis. J Radiol..

[9] Revel MP, Fournier LS, Hennebicque AS, Cuenod CA, Meyer G, Reynaud P (2002). Can CT replace bronchoscopy in the detection of the site and cause of bleeding in patients with large or massive hemoptysis?. Am J Roentgenol..

[10] McWilliams AM, Mayo JR, Ahn MI, MacDonald SLS, Lam SC (2006). Lung cancer screening using multi-slice thin-section computed tomography and autofluorescence bronchoscopy. J Thorac Oncol..

[11] Herth FJF, Eberhardt R, Anantham D, Gompelmann D, Zakaria MW, Ernst A (2009). Narrow-band imaging bronchoscopy increases the specificity of bronchoscopic early lung cancer detection. J Thorac Oncol..

[12] Thiberville L, Saiaiin M, Lachkar S, Dominique S, Moreno-Swirc S, Vever-Bizet C (2009). Human in vivo fluorescence microimaging of the alveolar ducts and sacs during bronchoscopy. Eur Respir J..

[13] Poe RH, Israel RH, Marin MG, Ortiz CR, Dale RC, Wahl GW (1988). Utility of fiberoptic bronchoscopy in patients with hemoptysis and a nonlocalizing chest roentgenogram. Chest..

[14] O'Neil KM, Lazarus AA (1991). Hemoptysis. Indications for bronchoscopy. Arch Intern Med..

[15] Set PA, Flower CD, Smith IE, Chan AP, Twentyman OP, Shneerson JM (1993). Hemoptysis: comparative study of the role of CT and fiberoptic bronchoscopy. Radiology..

[16] Thirumaran M, Sundar R, Sutcliffe IM, Currie DC (2009). Is investigation of patients with haemoptysis and normal chest radiograph justified?. Thorax..

[17] Tsoumakidou M, Chrysofakis G, Tsiligianni I, Maltezakis G, Siafakas NM, Tzanakis N (2006). A prospective analysis of 184 hemoptysis cases – diagnostic impact of chest X-Ray, computed tomography, bronchoscopy. Respiration..

[18] Danish Lung Cancer Group (DLCG) (2014). Guideline in lung cancer [Internet].

[19] Bønløkke S, Guldbrandt LM, Rasmussen TR (2015). Bronchoscopy in patients with haemoptysis and normal computed tomography of the chest is unlikely to result in significant findings. Dan Med J..

[20] Uzun O, Atasoy Y, Findik S, Atici AG, Erkan L (2010). A prospective evaluation of hemoptysis cases in a tertiary referral hospital. Clin Respir J..

